# Paralysis of the Arm Relieved

**Published:** 1845-10

**Authors:** 


*Paralysis of the arm relieved.*—On the 12th of March last, we were called to see a negro boy about ten years of age, the property of Miss D., and found him laboring under complete paralysis of the right arm. The boy's mother gave us the following history:

Two nights previous to our visit, something like a noise caused her to notice the boy: she found him somewhat stupid, partially blind, and unable to move the right arm. He remained in this condition until our visit, about forty hours after.

The patient then presented the following symptoms:—Stupor, such as to render it difficult to arouse him; pulse firm and slow; bowels costive; complains only of pain in the head. We bled him generally and locally; blistered the nape of the neck; ordered mercurial and aloetic purges—his bowels were all the time very difficult to move. This treatment was pursued ten or twelve days, without any improvement. We than put him under the operation of the electro-galvanic battery, the operation of which was confided to his young master, to be made daily.

April 12. I happened to be in the neighborhood, and called to see the boy. I found him almost entirely relieved: he could throw a stone about as far as he ever could, and the only difficulty was a little weakness of the arm. The use of the battery was shortly after discontinued.

June 17. The arm has been gradually improving, and there is now scarcely any difference between it and the other.—*Ibid.*

